# The correlate of disordered eating with body image dissatisfaction and psychological health of Chinese university students in Hong Kong and Mainland China

**DOI:** 10.1186/2050-2974-2-S1-O37

**Published:** 2014-11-24

**Authors:** Sau Fong Leung, Lili Ma

**Affiliations:** 1 The Hong Kong Polytechnic University, Hong Kong; 2 Capital Medical University, Beijing, China

## 

University students are a high risk population for eating disorder symptomatology. The risk can be related to increased demand for academic achievement, high stress and anxiety, dating relationships, peer influence, struggle with self-concept, and role and identity changes. Given the surging risk of eating disorders among university students, it is essential to identify the factors correlate with disordered eating and develop effective intervention strategies. This study examined Chinese university students' disordered eating, body image dissatisfaction and psychological health, and their associated relationships. The participants were recruited through personal approach at different university campuses and social network sampling using strategies, such as e-mails, facebook, whatsapp and electronic promotional posters.

521 university students from various institutions completed several online questionnaires including the EDE-Q, the SCOFF questionnaire, the Stunkard's Figure Rating Scale, the Body Shape Questionnaire and DASS-21 administered through a "Smart ehealth" website. The result showed that 4% participants were at high risk for eating disorders, many had body image dissatisfaction (81.6%), and some had symptoms of anxiety (41.7%), depression (27.4%) and stress (20.5%). Their eating disorder psychopathology was significantly correlated with their body shape dissatisfaction (r^2^=0.809, P<0.001) and psychological symptoms (r^2^=0.378, P<0.001) as revealed from the Spearman's Rank Order Correlation.

This abstract was presented in the **Prevention & Public Health** stream of the 2014 ANZAED Conference.

